# Optical characteristics of the skin with dark circles using pump-probe imaging

**DOI:** 10.1038/s41598-022-21131-5

**Published:** 2022-11-03

**Authors:** Yikang Hou, Xiaonan Yang, Lvping Huang, Zuoliang Qi, Ran Xiao

**Affiliations:** 1 Plastic Surgery Hospital, Chinese Academy of Medical Sciences and Peking Union Medical College, 33 Badachu Road, Beijing, 100144 China; 2 Bejing Tsinghua Changgung Hospital, School of Clinical Medicine, Tsinghua University, 168 Litang Road, Beijing, 102218 China

**Keywords:** Biological techniques, Biotechnology, Computational biology and bioinformatics, Medical research, Molecular medicine, Optics and photonics

## Abstract

Pump-probe imaging was first used for quantitative analysis of melanin in dark circles’ skin to improve the ability to diagnose and treat dark circles on human skin. This study aimed to compare the distribution characteristics in melanin of lower eyelid skin tissues and to determine whether pump-probe imaging has potential for the classification of dark circles in vivo. Specimens obtained from 15 patients undergoing blepharoplasty were examined using pump-probe imaging. Furthermore, adjacent slices were respectively treated with hematoxylin–eosin (HE) and ferrous sulfate (FeSO_4_) staining for cross-references. Subsequently, the melanin content index (MCI) and mean fluorescence intensity (MFI) were quantitatively analyzed by the pump-probe imaging. The distribution of melanin granules in the pump-probe image and FeSO_4_ staining was consistent. Meanwhile, the tissues of the skin with dark circles and normal skin demonstrated significant differences in MCI and MFI. These differences can be used to distinguish the skin with dark circles from the normal skin. Pump-probe imaging could be used for the analysis of the microstructure and spectral characteristics of melanin granules in skin with dark circles. Significant differences were noted between the pigmented type of dark circles and the other two groups (normal skin and the vascular type of dark circles), while no significant differences were found between normal skin and the vascular type of dark circles.

## Introduction

“Dark circles” is not an official medical term but a common condition that has been used to express the relative darkness of the skin around the eyes, which affects patients of all ages, especially women^1,2^. Dark circles around the eyes are not a health threat, but they can affect the quality of life and appearance, as they can make sufferers look tired or sad. Dark circles are caused by various factors, including infection, inflammation, allergies and some specific lifestyle (such as insufficient sleep, smoking and mental stress), which can lead to excessive pigmentation of the eyes, a common manifestation of dark circles^3–6^. At present, dark circles can be treated by various methods, such as normobaric oxygen therapy, depigmenting agents, chemical peeling, lasers, hyaluronic fillers, autologous fat transplantation and blepharoplasty^4,7–10^. Despite the multitude of treatment methods, the specific dose is not clear and accurate enough, and broadly applicable and effective treatments are lacking. Although dark circles are very common, no quantitative analysis was conducted on the severity of dark circles, and its pathogenesis is unclear^1,4,11,12^. In addition, currently, the lack of animal model to human dark circles influences the progress of experimental research on the pathophysiology of dark circles, which in turn hinders the development of clinical applications. In this study, a reliable noninvasive quantitative method will enable direct in vivo studies of human dark circles, the evolution of dark circles, and the pathophysiology of dark circles, without relying on invasive procedures in animal models.

Multiphoton microscopy based on pump-probe imaging is one of the most important inventions in the field of label-free optical imaging in recent years^13^. By taking advantage of high molecular selectivity and spatial resolution, it has become a powerful tool for disease identification, noninvasive assessment, and monitoring of the morphological and functional status of live tissues^14,15^. Pump-probe microscopy uses two ultrafast laser pulses (pump and probe) to study the transient excited and ground-state photodynamics of pigment molecules^16,17^. Although melanin is a complex biopolymer, its optical properties make it a potential biomarker under pump-probe imaging^18–20^. Pump-probe imaging of melanin has been applied in melanoma diagnosis^21^, metastasis analysis of melanoma^22^, and differential diagnosis between melanoma and melanin nevus^23^. However, it is rarely applied in skin pigmentation disease^16^, and there are no relevant reports on melanin research in dark circles.

According to literature reports^3,24^, We classified the eye bag skin of the enrolled patients and performed consistency analysis of pump-probe images and ferrous sulfate (FeSO_4_)-stained images of melanin in the lower eyelid skin. These two imaging methods of melanin were compared based on the intraclass correlation coefficient (ICC). Subsequently, the distribution of melanin granules was investigated, and the melanin content index (MCI) and the mean fluorescence intensity (MFI) were quantitatively analyzed simultaneously to determine whether the method can be used to distinguish between dark circles and normal skin and to evaluate its potential for noninvasive in vivo pathophysiological analysis of melanin, precision treatment of dark circles, and response to treatment.

## Materials and methods

### Specimens

In vitro, human specimens were taken from 15 female patients who received blepharoplasty, between age 30 and 65 years. A Fitzpatrick skin type III/IV^25^ and history of dark circles for at least 1 year were required to participate in this study. The exclusion criteria were any signs of infection or inflammation around the eyes, pregnancy, use of nonsteroidal anti-inflammatory drugs within 1 week, exposure to ultraviolet radiation within 1 month, and periocular injections or surgery within 1 year. All patients were from the Plastic Surgery Hospital, Chinese Academy of Medical Sciences, and Peking Union Medical College. Based on the physician’s visual inspection, three patients had normal skin, six showed purple shadowing of the lower eyelid skin (vascular type of dark circles), and another six mainly manifested dark pigmentation around the eyes (pigmented type of dark circles). Chinese Academy of Medical Sciences, Plastic Surgery Hospital Ethics Committee approved the experiments and the relevant details. All experiments were performed in accordance with the ethical requirements and institutional rules of human clinical research. All participants involved in the study provided informed consent.

Tissue slices were obtained from various lower eyelid skin including normal skin (n = 3), vascular type of dark circles (n = 6), and pigmented type of dark circles (n = 6). After the tissue sample was removed from the patient’s skin, it was immediately frozen and stored in − 196 °C liquid nitrogen until it was used. The tissue sections were cut into 6-mm thick sections, and each patient’s lower eyelid skin tissue had eight continuous slices. FeSO_4_ staining and pump-probe imaging were performed at intervals. One was not stained for nonlinear spectroscopy, sandwiched between the microscope slides and the cover slide, and the adjacent one was subjected to FeSO_4_ staining. And a pair of adjacent slices were selected for statistical analysis. This study analyzed a total of 30 tissue slices across all 15 patients. To avoid dehydration or contraction during imaging, a small amount of phosphate-buffered saline solution was dripped in tissue specimens. Solution samples of melanin (Yuanye Bio-Technology, Shanghai, China) were prepared according to established methods at a concentration of 0.84 mg/mL^23^.

### Spectral imaging system

**Figure 1:**
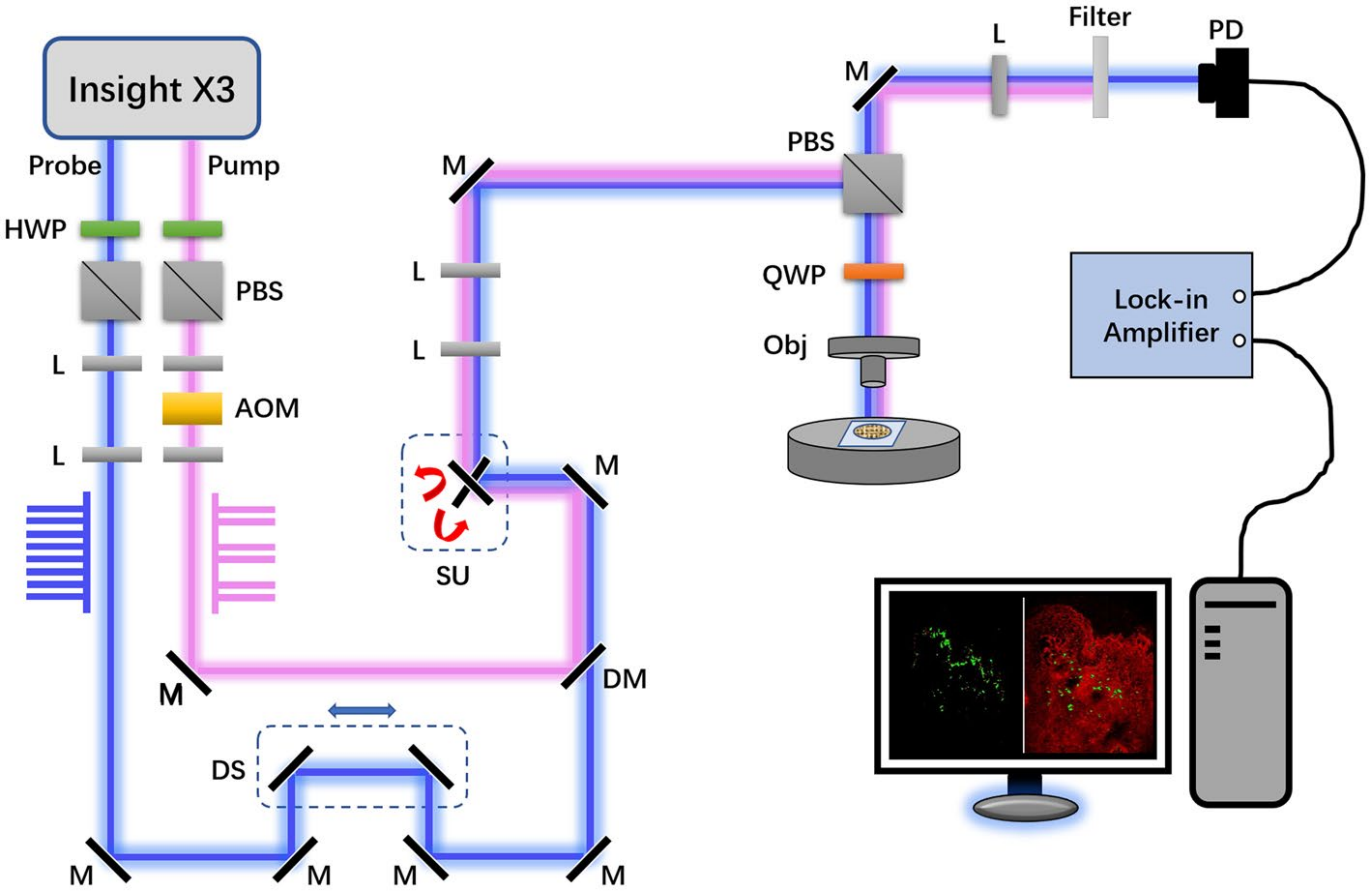
Schematic design of pump-probe imaging system. *HWP* half-wave plate, *PBS* polarizing beam splitter, *L* lens, *AOM* acousto-optic modulator, *M* mirror, *DS* delay stage, *DM* dichroic mirror, *SU* scanning unit, *QWP* quarter-wave plate, *Obj* objective, *PD* photodiode.

A dual-output fs laser system (InSight X3, Spectra-Physics, Newport) providing two phase-locked fs lasers with an 80-MHz repetition rate was employed for transient absorption imaging. One 120-fs laser with 850-nm wavelength was used as the probe beam. The other 200-fs laser centered at 1045 nm, which served as the pump beam, was modulated by an acoustic–optic modulator (1205-C, Isomet) at ~ 2.8 MHz. The two beams were collinearly combined through a dichroic mirror (DMSP950, Thorlabs). A 40 × water immersion objective (NA = 0.8, UPlanApo, Olympus) was used to focus the light on the sample, and an oil condenser (NA = 1.4, U-AAC, Olympus) was used to collect the laser from the sample. Two filters (ET845/55 m, ET795/150 m, Chroma) were used to filter out the pump beam, the probe beam was detected by a photodiode, and the probe beam loss signal was extracted by a lock-in amplifier (HF2LI, Zurich Instrument). Schematic design was shown in Fig. 1.

### Statistical analysis

Using the image analysis program (Image J, National Institutes of Health, USA), we calculated the MCIs of pump-probe images and FeSO_4_-stained images (200 ×), and the ICC of the two MCIs were compared. In the Image J software, the pump-probe image and FeSO_4_ stained image of the lower eyelid skin tissue sample were modified to 8-bit mode, and then the threshold was adjusted to ensure that all of the entire target areas (melanin particles) were selected. Calculated the percentage of the melanin particles area (%Area) and defined it as the melanin content index (MCI). Then, the relative relationship between the two MCIs and the L* value of the corresponding patient’s standard photograph was also analyzed. The L* values were derived from the red, green, and blue values in the Lab mode of the image. The lower the L* value is, the darker the color of the target area is. In the pump-probe image, five random regions of the epidermal basal layer were selected, and the mean value of the percentage of the melanin area to the region’s total area (MCI-1) and the mean fluorescence intensity of the region (MFI-1) were measured. When MCI was obtained, the fluorescence intensity of melanin granules in this area could also be obtained. Then selected five target areas of the same size and calculated their fluorescence intensity and the mean value, that is, the mean fluorescent strength (MFI). Similarly, five dermal layer regions were selected, and MCI-2 and MFI-2 of the target regions were measured. Finally, data analysis and processing were performed using SPSS for Windows version 26.0 (IBM Corp, Armonk, NY, USA.), and P < 0.05 was considered different.

## Results

### Different types of dark circles and their hematoxylin–eosin (HE) and FeSO_4_ staining images

**Figure 2:**
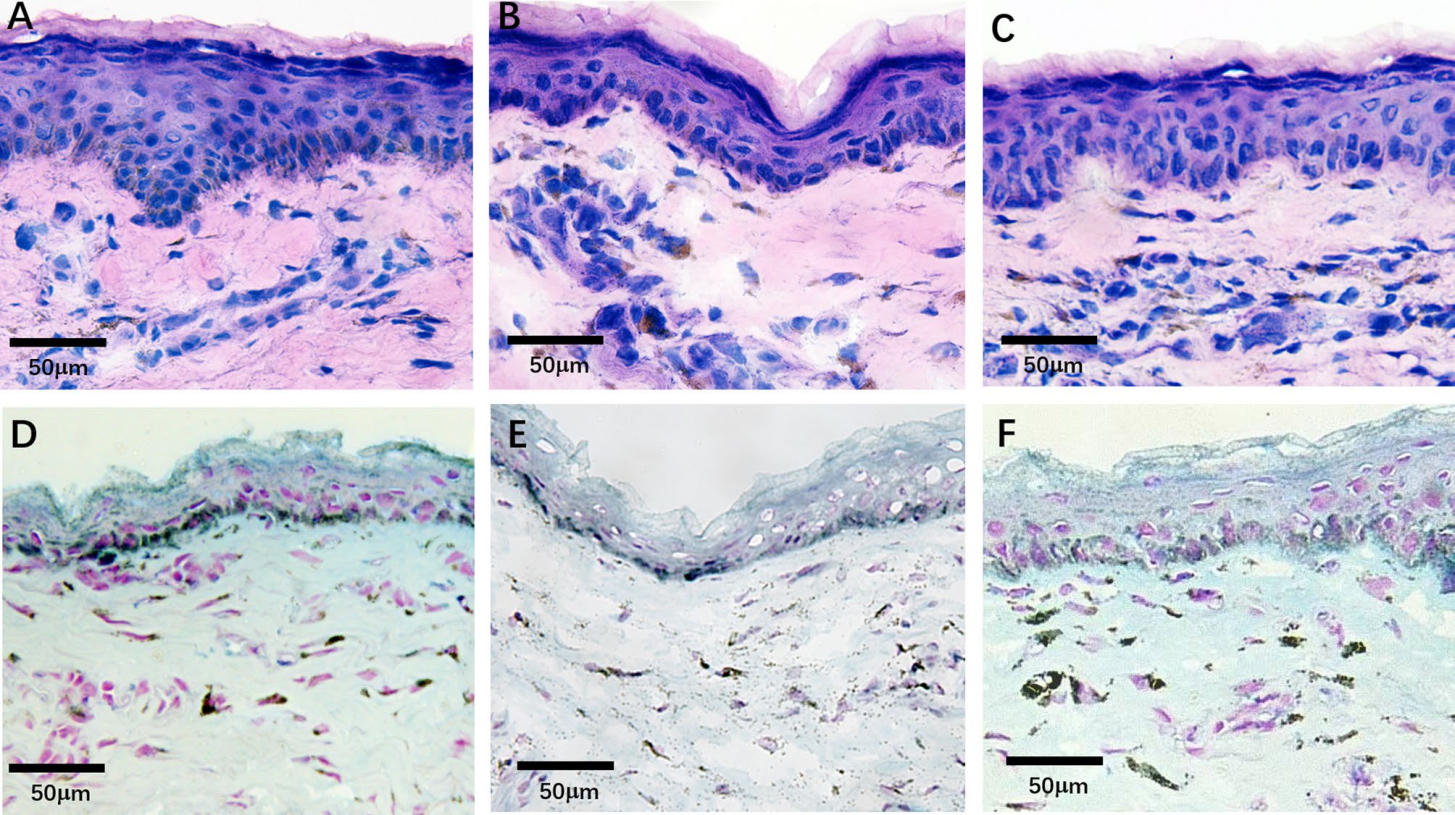
HE and FeSO4 staining images in different types of dark circles. (**A**) HE staining of normal skin. (**B**) HE staining of skins with vascular type of dark circles. (**C**) HE staining of skins with pigmented type of dark circles. (**D**) FeSO4 staining of normal skin. (**E**) FeSO4 staining of skins with vascular type of dark circles. (**F**) FeSO4 staining of skins with pigmented type of dark circles.

Unstained slices from a set of 15 patients who had undergone blepharoplasty were examined by pump-probe imaging and spectroscopy. As shown in HE staining, it is nearly impossible to find stained melanin granules in the slice of the normal lower eyelid skin (Fig. 2A) and small amounts of melanin granules scattered within macrophages in the dermis of the vascular group (Fig. 2B). The density and size of melanin granules in the pigmented group were significantly higher than those in the normal and vascular groups, but the difference between the latter two were not significant (Fig. 2A–C). Similar performance could also be seen in FeSO_4_ staining (Fig. 2D–F). However, melanin staining result in FeSO_4_ staining was clearer than that of HE staining.

### Pump-probe imaging of melanin in lower eyelid skin samples

**Figure 3:**
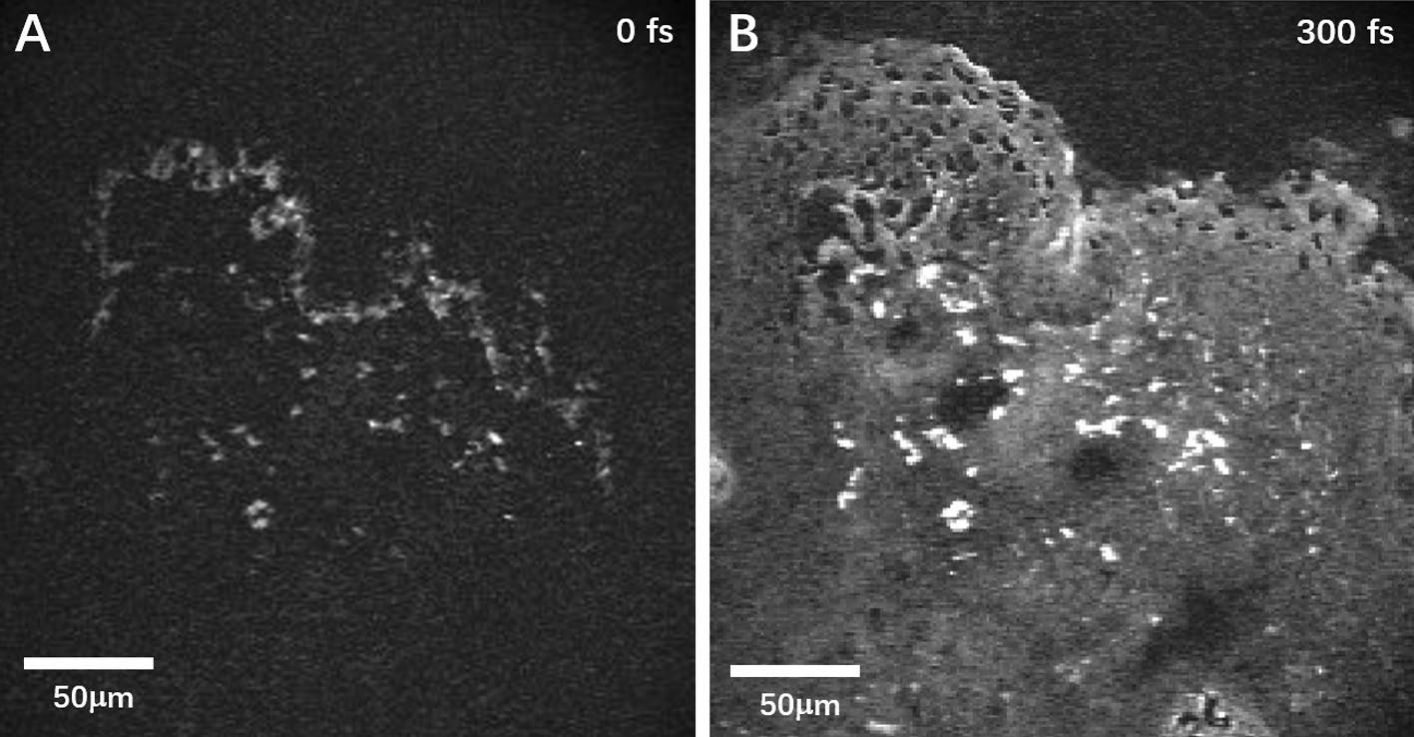
Pump-probe raw images of the lower eyelid skin section. (**A**) Pulse interval 0 fs. (**B**) Pulse interval 300 fs.

**Figure 4:**
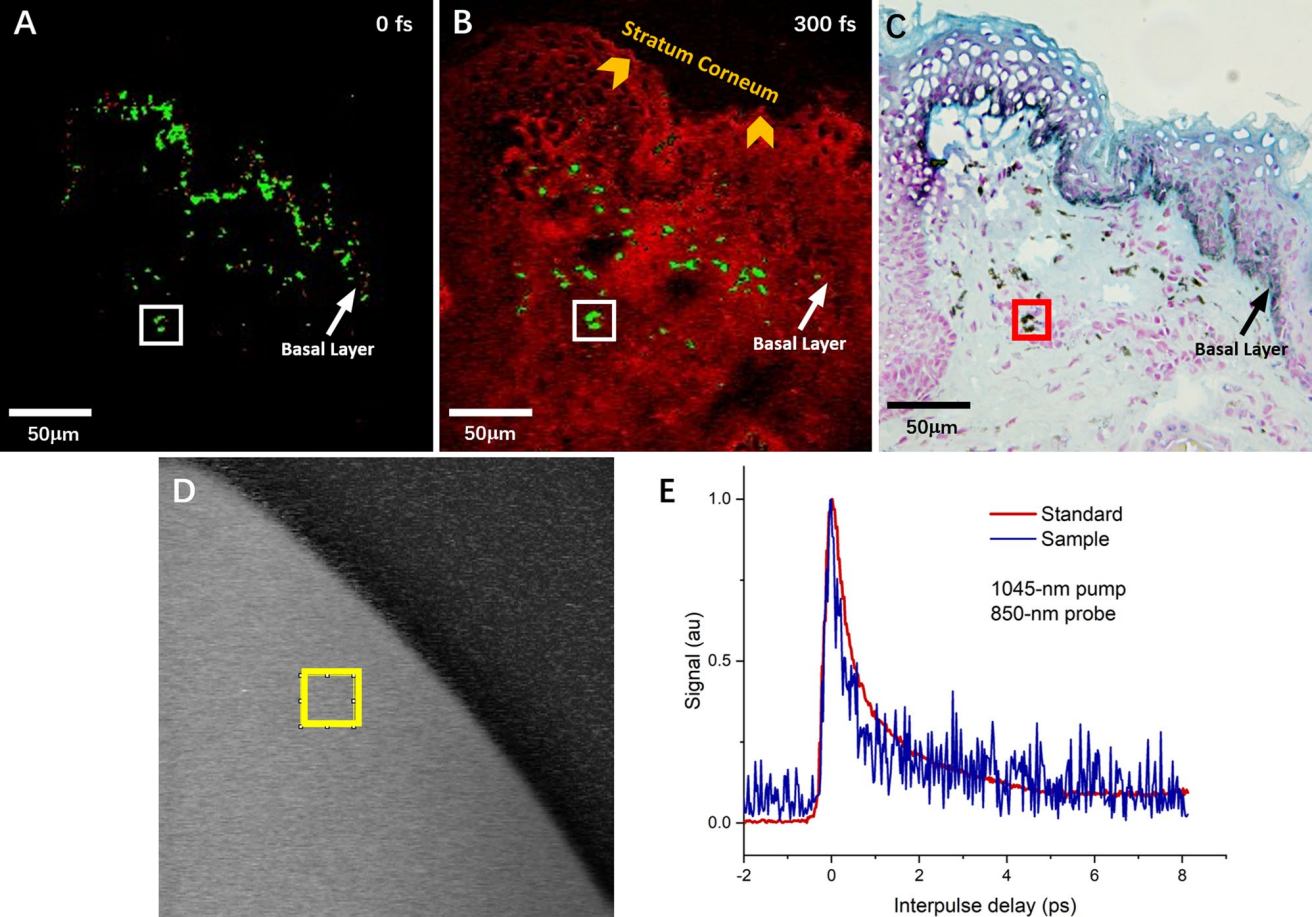
Pump-probe signal and FeSO4 staining of the same field in the lower eyelid skin section of subject 4. (**A**) Pump-probe image with pulse interval 0 fs. (**B**) Pulse interval 300 fs. (**C**) FeSO_4_ staining of the same position. (**D**) A target region (yellow box) in the transient absorption signal of pure melanin solution was randomly selected for spectrum analysis. (**E**) Similar pump-probe time delay traces between tissue regions of white boxes in (**A**) and (**B**) with the target region of pure solution melanin (yellow box in **D**).

The slices were imaged with multiphoton scanning laser microscopy, with a laboratory designed microscope using 35 mW of 1045-nm pump light, 20 mW of 850-nm probe light, and 40 × 0.8 numerical aperture (NA) objective, and the transmitted probe light was collected (Fig. 3A,B). Pump-probe images were modified to 8-bit mode, and then the threshold was adjusted to ensure that all of the entire target areas (melanin granules) were selected (Fig. 4A,B). Pump-probe images were compared with FeSO_4_-stained images to confirm the source of the signal as melanin granules (Fig. 4). It revealed the same precise details in the distribution of melanin granules as the bright-field images of a similar field in Fig. 4C. At zero delays between the pump and the probe pulse, the base cell layer separated the epidermal layer and the dermis, and the basal layer was easily identified as thick-bright melanin dots, with a typical papillary protrusion (Fig. 4A). When the interpulse delay was increased to 300 fs to remove the contribution of any coherent or instantaneous effects, the stratum corneum is exposed, which could be represented as a bright line due to the strong signal of the tissue edge. The dermis could also show scattered melanin granules, which demonstrated a positive signal from real excited states generated by the pump pulse (Fig. 4B, white box). Meanwhile, the pure melanin solution showed a uniform gray bright signal in the pump-probe imaging (Fig. 4D, yellow box). By analyzing the spectra of the target signal points in pump-probe images of the label-free sections (Fig. 4A,B, white box) and standard substance (pure melanin solution) (Fig. 4D, yellow box), the signal curve behavior of the label-free sections is almost consistent with that of the standard substance (Fig. 4E).

**Figure 5:**
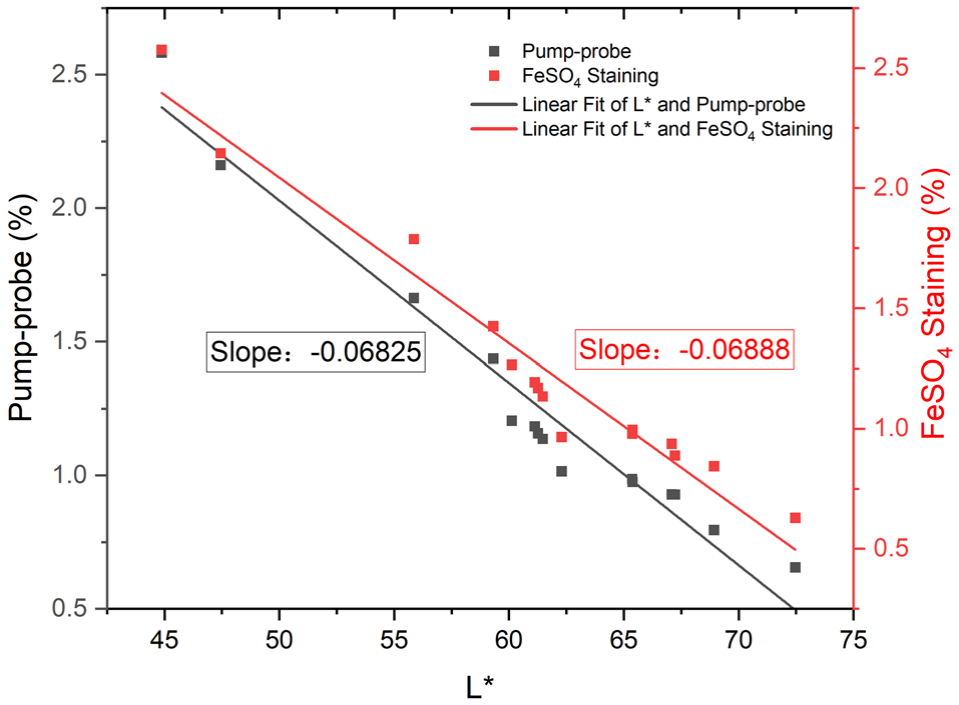
Correlation between the MCI and the L* value.

Then, we calculated the area fractions of melanin granules in pump-probe images and FeSO_4_-stained images, which were named as MCI (Fig. 5). The MCIs of two sorts of images were negatively correlated with the L* value of every patient’s photograph under standard conditions, while the linear fit of L* and MCI in pump-probe images showed nearly the same slope as that in FeSO_4_ staining (Fig. 5). In addition, the ICC of MCIs in the two imaging methods was 0.997 (P < 0.001).

### Quantization comparison of melanin in the skin with dark circles and normal skin

**Figure 6:**
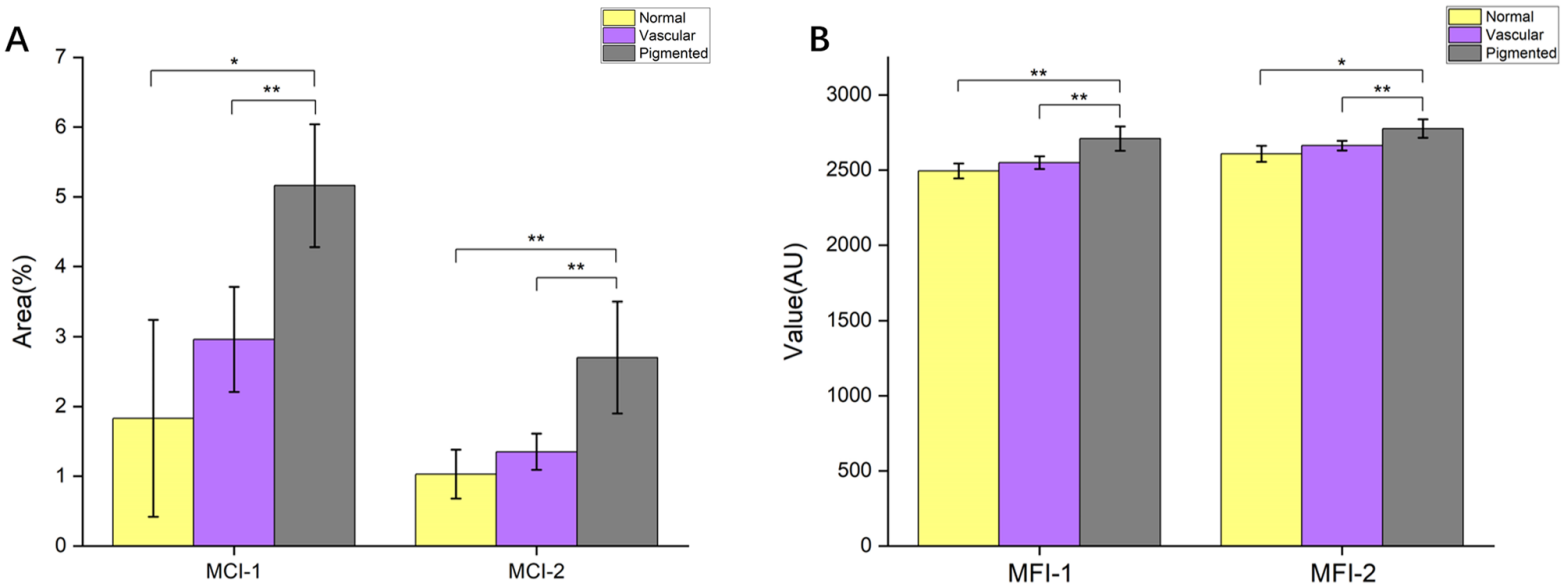
MCIs and MFIs of different groups in pump-probe imaging. (**A**) MCI of different groups. (**B**) MFI of different groups. (*P < 0.05; **P < 0.01).

Randomly and respectively, five regions of the epidermal basal layer and five dermal regions were selected to calculate the MCI-1 and MCI-2. In the comparison of the pigmented group with the vascular group and normal group, MCI-1, and MCI-2 values were significantly elevated and different (Fig. 6A). However, the MCI-1 and MCI-2 of the vascular group were also higher than those of the normal group, but the differences were not significant (Fig. 6A). The same results were observed in MFIs, which contained MFI-1 in the epidermal basal layer and MFI-2 in the dermal region as well, with significantly higher values in the pigmented group than in the vascular group and normal group, but no significant difference was found between the latter two groups (Fig. 6B). All the data and details of patients were shown in Table 1.

**Table 1:** Details and data of all subjects.

Subject	Group	Age	L*	FeSO_4_	Pump-probe				
					MCI	MCI-1	MFI-1	MCI-2	MFI-2
1	Vascular	39	67.09	1.13	1.14	3.65	2580.75	1.52	2691.69
2	Pigmented	42	47.44	2.15	2.16	5.63	2768.06	2.21	2785.22
3	Vascular	37	65.36	0.89	0.93	2.53	2518.13	1.19	2640.27
4	Pigmented	56	44.88	2.58	2.58	6.75	2825.25	3.95	2889.58
5	Pigmented	42	62.30	1.19	1.18	4.43	2635.30	1.88	2719.79
6	Vascular	37	59.31	1.17	1.16	3.68	2612.34	1.06	2638.75
7	Pigmented	50	61.13	1.27	1.21	4.62	2640.27	3.39	2725.05
8	Vascular	34	68.93	0.98	0.98	2.75	2541.55	1.25	2643.11
9	Normal	59	72.47	0.63	0.66	0.65	2449.63	0.78	2553.60
10	Vascular	38	61.47	1.00	0.99	3.38	2546.36	1.32	2648.25
11	Normal	49	67.23	0.97	1.02	3.39	2547.58	1.43	2660.96
12	Vascular	45	61.27	0.94	0.93	1.78	2493.38	1.77	2713.78
13	Pigmented	46	55.86	1.79	1.66	4.79	2739.83	2.24	2762.05
14	Pigmented	62	60.12	1.43	1.44	4.76	2644.80	2.55	2774.20
15	Normal	42	65.383	0.844	0.795	1.46	2484.8426	0.89	2609.18

*MCI* the melanin content index, *MCI-1* MCI of the epidermal basal layer, *MCI-2* MCI of the dermal layer, *MFI-1* the mean fluorescence intensity of the epidermal basal layer, *MFI-2* the mean fluorescence intensity of the dermal layer.

## Discussion

In the human skin, the distribution and content of melanin in the epidermis and dermis play important roles in determining the color of the human skin^26^. After being produced by melanocytes, melanin granules mainly aggregate at the dermal–epidermal junction (DEJ) and disperse in dermal macrophages^27^. Both HE staining and FeSO_4_ staining required biopsy and are time-consuming. Therefore, in the field of melanin imaging, especially in the quantitative analysis of melanin granules in skin with dark circles, it is very necessary to apply a noninvasive, accurate, fast and label-free method.

Pump-probe imaging allows specific imaging of pigment molecules and is characterized by low tissue damage and strong penetration depth^28^. Specifically, in the pump-probe imaging in this study, melanin in dispersed macrophages tends to lack negative signal at zero delay, which may be due to the thermal effects of concentrated melanin absorbing the laser and dissipating in the form of heat or the influence of oxidative degradation^28^. These results were consistent with FeSO_4_ staining of melanin, which suggests that pump-probe imaging can be used for melanin imaging in skin tissues of the lower eyelid, distinguishing melanin between epidermal cells and macrophages, and even helping distinguish macrophages and melanocytes in vivo^28^. Under the same imaging conditions (pump, 1045 nm; probe, 850 nm), which shielded the influence of the Raman spectrum of melanin, the transient absorption spectrum of the lower eyelid skin (Sample) was very similar to that of pure melanin solution (Standard), which further confirmed that the target regions we selected in the sample were indeed melanin granules.

In terms of correlation analysis, the MCIs of the two melanin imaging methods (pump-probe imaging and FeSO_4_ staining) showed a high consistency, indicating that the pump-probe imaging method is reliable for the quantitative analysis of melanin. In addition, the MCIs of both melanin imaging methods showed a negative correlation with the L* value, which means that the darker the skin of the lower eyelid, i.e. the lower L*, the higher the proportion of melanin in pump-probe and FeSO_4_ staining. Moreover, the linear fit of L* and pump-probe imaging showed nearly the same slope with the linear fit of L* and FeSO_4_ staining, which further indicates that the pump-probe method has high reproducibility in melanin imaging and high accuracy in the quantitative analysis of melanin. This is an interesting observation because, so far, only a few studies have examined the relationship between the quantitative index of melanin and the L* value.

Pump-probe imaging and statistical analysis revealed that the density of melanin granules in the pigmented group was significantly higher than that in the normal group and the vascular group (in both epidermis and dermis), which as Watanabe et al.^29^ found by anti-S100 protein and Masson-Fontana silver staining, periorbital pigmentation was mainly characterized by a relative increase in the number of skin melanocytes and melanin content. In addition, when there is little or no subcutaneous tissue, the color of the orbicularis oculi muscle will be revealed, or the number of microvessels in the skin of the lower eyelid is higher than that in the cheeks, which will cause the purple shadowing of the skin of the lower eyelid^4,30^. This is because the skin in the vascular group is thinner than that in the normal group; thus, melanin is more easily deposited and the MCI-1 and MCI-2 values were slightly higher than those of the normal group.

In pump-probe imaging, the MFI of melanin granules in the pigmented group was significantly higher than that in the normal group and vascular group. The main reason is the significantly increased melanin content of the pigmented group when compared with the other two groups. Therefore, significant differences were noted in the number of photons emitted by the instantaneous absorption of the melanin in a certain area of pump-probe imaging. Interestingly, the difference in MFI-1 and MFI-2 between groups is consistent with that in MCI-1 and MCI-2, as Antoniou et al.^31^ used multiphoton tomography to observe different skin depths and types of melanin, that is, fluorescence intensity is correlated with melanin concentration. In summary, MCI and MFI are reliable indicators of identifying the difference between the skin with dark circles and normal skin, assessing treatment response, and tracking the development of dark circles.

The advantages of abundant molecular contrast signals and high spatial resolution make pump-probe microscopic imaging an ideal method to investigate melanocytosis^15^. Multiple microscopy methods have been used for the analysis of melanin content (such as diffuse reflectance spectroscopy, optical coherence tomography and confocal microscopy). However, none of the above methods can distinguish different melanin species. Pump-probe microscopy, on the other hand, takes advantage of the difference in electron transfer rate within melanin molecules, which is the only method that can specifically distinguish different species. Although this study did not use the distortion of species, this method is the best means to study melanin.

In this study, pump-probe imaging was used in evaluating dark circles for the first time, and the results confirmed the linear correlation between the pump-probe imaging and the L* value. However, the L* value needs to be obtained under strict and unified photographic conditions, and relevant software was required for correction. In addition, the results of this study confirmed that pump-probe imaging could accurately analyze the melanin content and fluorescence intensity in dark circles. Not only did the imaging conditions stable with very few interference factors, but also could characterize melanin of different depths, which could guide the depth of clinical treatment (such as choosing a laser with appropriate depth and model). Therefore, the results of this study provide some insights into the study of melanin deposition-related diseases in plastic surgery and dermatology.

**Figure 7:**
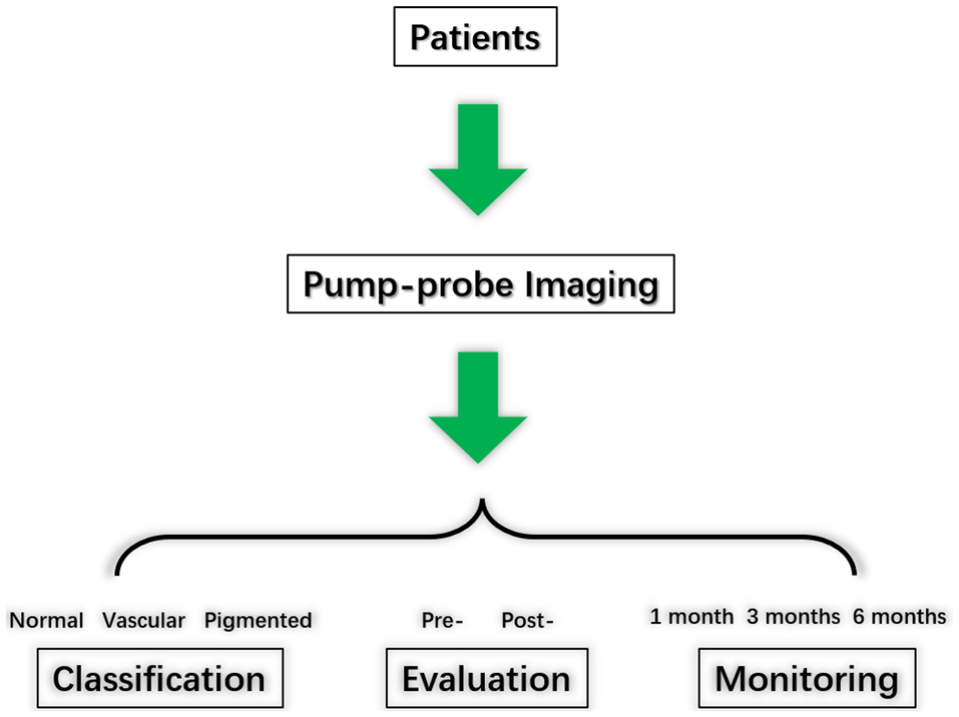
In vivo pump-probe imaging and classification-evaluation-monitoring system.

This study aimed to verify the feasibility of pump-probe imaging for quantitative analysis of the melanin granules in the lower eyelid skin through ex vivo experiments. The experimental results confirmed the accuracy of pump-probe imaging in melanin and the possibility of classification in dark circles. In terms of clinical applications, only certain parameters need to be adjusted based on the current imaging system and the corresponding adapter at different parts of the body needs to be developed, the non-invasive application of pump-probe imaging could be theoretically implemented. Figure 7 shows the clinical application of in vivo pump-probe imaging and classification-evaluation-monitoring system. This technology could characterize melanin of different depths, which could guide the depth of clinical treatment, such as choosing a laser with an appropriate model. Therefore, this study laid the foundation for the application of pump-probe imaging in the diagnosis and treatment of dark circles. With the development and diversification of hyperspectral imaging instrument miniaturization, clinicians may then classify skin disorders, evaluate treatment responses, and monitor disease progression based on pump-probe images of pigmentation lesions.

## Conclusion

In this study, we describe a pump-probe imaging method for the analysis of the microstructure and spectral characteristics of melanin granules in skin with dark circles. We demonstrate that pump-probe imaging can distinguish skin with dark circles from the normal skin. We obtained some interesting results: (i) Melanin showed consistent distribution characteristics on pump-probe imaging and FeSO_4_-staining, with aggregation along the basal layer of the epidermis and dispersion in macrophages in the superficial dermis. (ii) Pump-probe imaging and FeSO_4_ staining demonstrated a high ICC of 0.997 (P < 0.001), and the MCI of both types of melanin imaging showed a negative correlation with the L* value of the lower eyelid skin. (iii) Significant differences were noted in the melanin content index and the mean fluorescence intensity of melanin granules between the pigmented group and the other two groups, while no significant differences were found between the vascular group and the normal skin. With the development of hyperspectral imaging instrument miniaturization, we believe that pump-probe imaging can be a valuable tool for noninvasive in vivo evaluation of dark circles or other pigmentation lesions research and monitoring the development of diseases.

## Data Availability

The data that support the findings of this study are openly available in Mendeley Data (Hou, Yikang (2022), “Optical Characteristics of the Skin with Dark Circles Using Pump-Probe Imaging”, Mendeley Data, V1, https://doi.org/10.17632/7m2ydnysg5.1).
